# Tissue plasminogen activator (tPA) attenuates propofol-induced apoptosis in developing hippocampal neurons

**DOI:** 10.1186/s40064-016-2091-y

**Published:** 2016-04-18

**Authors:** Chao Liang, Ming Ding, Fang Du, Jing Cang, Zhanggang Xue

**Affiliations:** Department of Anesthesiology, Zhongshan Hospital, Fudan University, Fenglin Road 180, Shanghai, 200032 China

**Keywords:** Tissue plasminogen activator, Propofol, Apoptosis, Developing neurons

## Abstract

**Background:**

We investigated the effect of propofol on the tissue plasminogen activator (tPA) release in developing hippocampal neurons, and explored the effects of exogenous tPA on the propofol-induced neuron apoptosis.

**Methods:**

Primary hippocampal neurons isolated from neonatal Sprague-Dawley rats were exposed to propofol (20, 50, and 100 μM) for 6 h either one time or three times. Finally, neurons were pretreated with exogenous tPA (5 µg/ml), followed by propofol exposure (100 μM, 6 h). The neuron apoptosis was detected by terminal transferase deoxyuridine triphosphate-biotin nick-end labeling (TUNEL) and the protein expression of cleaved caspase-3 (Cl-Csp3) was analyzed by western blot, the tPA in media was tested by enzyme-linked immunosorbent assay.

**Results:**

Propofol exposure significantly increased the number of TUNEL-positive neurons and Cl-Csp3 expression in developing hippocampal neurons. Propofol decreased tPA level in the media of developing hippocampal neurons. The neuron appotosis induced by propofol was attenuated by pretreatment of tPA.

**Conclusion:**

Propofol exposure decreased tPA release in developing hippocampal neurons. The addition of tPA could partially reverse the apoptotic effect of propofol.

## Background

Propofol administered during synaptogenesis can produce long-term neurobehavioral and functional deficits in animals (Creeley et al. 2013; Cattano et al. 2008; Yu et al. 2013; Li et al. 2014; Xiong et al. 2014), since the developing brain is more vulnerable to anesthetic-induced neurotoxicity (Cattano et al. 2008; Jevtovic-Todorovic et al. 2003; Fredriksson et al. 2007; Ikonomidou et al. 1999). It has been demonstrated recently that proBDNF–p75NTR signaling pathway is the key player of propofol and isoflurane-induced neurotoxicity (Head et al. 2009; Lemkuil et al. 2011; Pearn et al. 2012).

The brain-derived neurotrophic factor (BDNF) is stored as a proneurotrophin (proBDNF) within synaptic vesicles and is proteolytically cleaved to mature BDNF (mBDNF) in the synaptic cleft by plasmin (Lee et al. 2001; Keifer et al. 2009; Lu et al. 2006). The mBDNF bind with the receptor kinase B and leads to neurite outgrowth and synapse stabilization and maturation (Lee et al. 2001; Keifer et al. 2009; Lu et al. 2005).While the proBDNF binds to the p75 neurotrophin receptor (p75^NTR^) and initiates cellular processes that inhibit axonal elongation and cause growth cone collapse and apoptosis (Huettner and Baughman 1986). The number of plasmin in the synaptic cleft is mainly depending on the tissue plasminogen activator (tPA), which convert the plasminogen into plasmin. When the tPA is decreased, the convertion of plasminogen into plasmin may be blunted, and the number of proBDNF is increased, which then leads to inhibition of axonal elongation, growth cone collapse, and apoptosis.

The release of tPA from presynaptic vesicles is activity-dependent (Lemkuil et al. 2011), and it is therefore possible that anesthetics might suppress neuronal activity, reduce tPA release which finally results in the proBDNF-mediated neuron growth inhibition pathway dominant, leading to the final neuronal apoptosis. However, it is still unknown that the direct effect of propofol on the release of tPA in developing neurons, and more importantly, whether the addition of exogenous tPA could reverse the propofol induced neurotoxicity has not been investigated.

We examined whether propofol decreases tPA release, which leads to decrease proBDNF, in hippocampal neurons. In addition, we examined whether the addition of exogenous tPA attenuates the propopol-induced apoptosis in hippocampal neurons.

## Methods

The experimental protocols were approved by the animal experimental ethics committee of Zhongshan Hospital, Fudan University. All experimental procedures were performed in accordance with the Guidelines for the Care and Use of Experimental Animals. Hippocampal neurons of neonatal Sprague-Dawley rats were isolated by using the method described previously. Neurons were isolated from the postnatal day 1 or 2 pups and grown in culture for 4–6 days in vitro. Neurons were cultured in media (Neuobasal A) supplemented with 250 mM GLUTMax1 (Santa Cruz, CA), B27 (2 %), and penicillin/streptomycin (1 %). Poly-d-lysine/laminin (2 g/cm^2^) coated 48-well plates were used to culture neurons at 37 °C in 5 % CO_2_ before experiments.

### Cell culture and treatment

The cultures were placed within an incubator (a gas mixture of 5 % CO_2_, 21 % O_2_, balance nitrogen at a flow rate of 2 l/min, 37 °C) and exposed to propofol (20, 50, and 100 μM) for 6 h either one time or three times (once per day for 3 consecutive days). In the multiple exposure groups, the neurons were rinsed with PBS for one time after each exposure, and placed in fresh media overnight. Finally, neurons were pretreated by recombinant human tPA (Santa Cruz, CA) at different concentration (0.05–5 μg/ml) for 15 min, followed by propofol 100 μM exposure for 6 h. After respective treatments, the neurons were harvested and subjected to the following measurements.

### TUNEL staining

After each treatment, DNA fragmentation was detected for apoptotic neurons by using the fluorometric TUNEL system (Roche Nutley, NJ) According to the manufacturer’s instructions, neurons were fixed in 4 % paraformaldehyde in PBS at room temperature for 20 min, incubated with fluorescein-conjugated TdT enzyme at 37 °C for 1 h in humidifying chamber, and then mounted with DAPI (4′,6′-diamidino-2-phenylindole) for nuclear counter staining. Both TUNEL-and DAPI-positive neurons were counted by using a Nikon Eclipse 80i fluorescence microscope (Nikon, Tokyo, Japan). Apoptosis rate was quantified by determining the ratio of TUNEL-positive nuclei to total cell nuclei.

### tPA ELISA

In brief, after treatment by propofol, media from neurons were frozen at −80 °C before preforming enzyme-linked immunosorbent assay (ELISA) (Invitrogen, Carlsbad, CA). A 48-well plate was precoated with biotinylated plasminogen activator inhibitor-1 for 30 min, washed three times. The tPA standard (0.05–10 ng/ml) and tested samples were added to the plate for 30 min. After incubated with anti-tPA primary antibody and anti-rabbit horseradish peroxidase-conjugated secondary antibody for 30 min, respectively, the tetramethylbenzidine substrate was added, and the reaction was quenched 10 min later by 1 M H_2_SO_4_ and read at 450 nm on a spectrophotometer (TECAN Infinite M200, San Jose, CA).

### Western blot

Briefly, after respective treatments, neurons were lysed and 15 μg of proteins were boiled in a sample buffer (50 mM Tris–HCl pH 7.5, 150 mM NaCl, 1 % SDS, 100 mg/mL PMSF) and then separated on 15 % SDS-PAGE gels. The separated proteins were transferred to a nitrocellurose membranes (CNI, Canada), which was subsequently blocked in Tris-buffered saline-Tween 20 (TBST) containing 5 % (w/v) nonfat dried milk for 1 h at room temperature. The membrane was then incubated with appropriate concentration of primary antibody of Cl-Csp3 (1:1000, Vector Laboratories, Burlingame, CA) at 4 °C overnight. The membrane was washed and subsequently probed with horseradish per-oxidase-conjugated secondary antibody (1:2000, Sigma-Aldrich, St. Louis, MO). Blots were finally developed with a chemiluminescent HRP substrate kit (Sigma-Aldrich, St. Louis, MO, USA) and all protein bands were quantitated with a ImageJ version 1.38 (NIH, Bethesda, MD).

### Statistical analysis

SPSS16.0 software was used to perform statistical analyses of the data. Statistics were performed using one way ANOVA and Student′s *t* test. All results are expressed as the mean ± SD, and *P* *<* 0.05 was considered statistically significant.

## Results

### The effects of propofol on neuron apoptosis

**Fig. 1:**
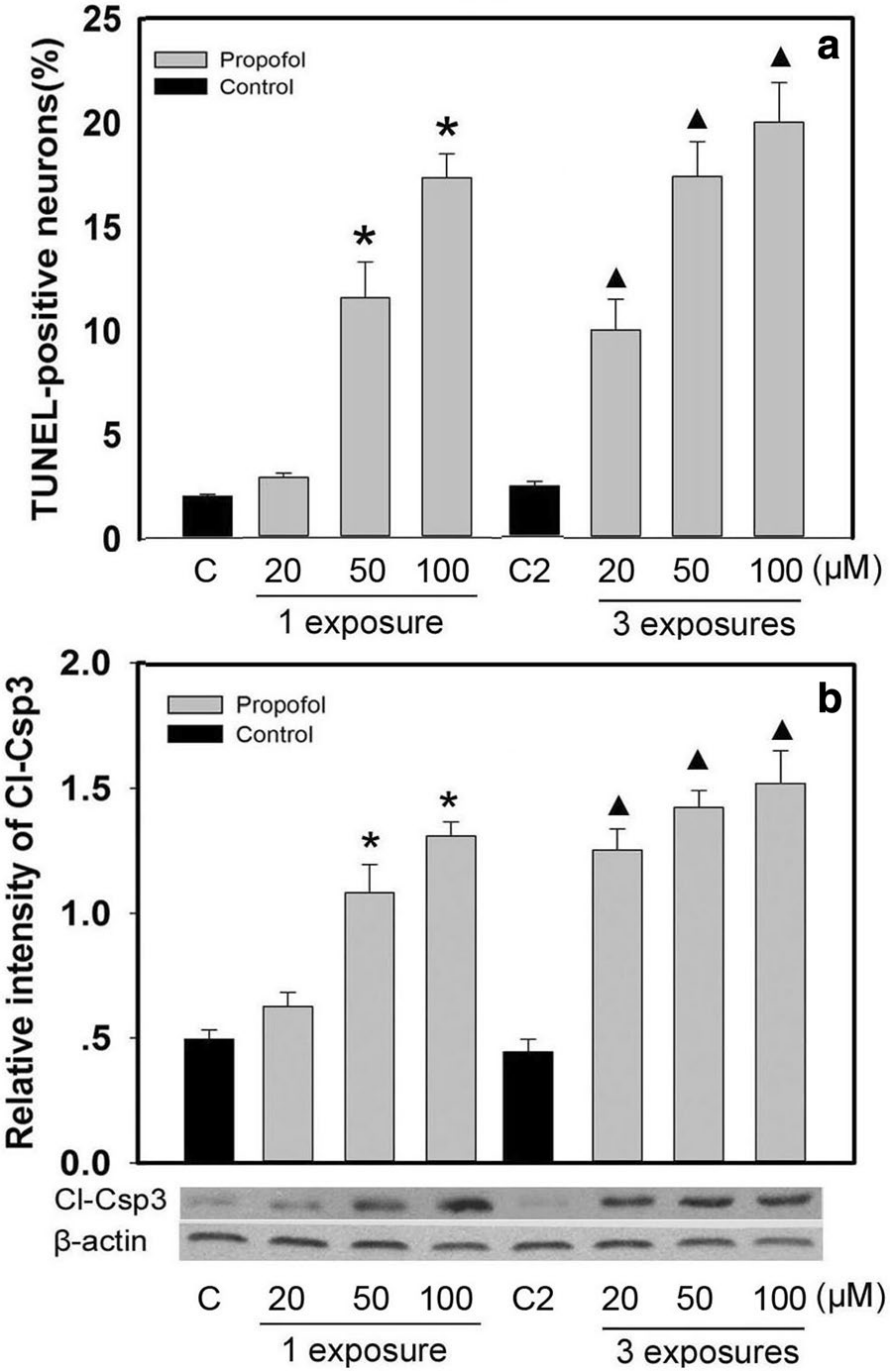
Propofol induces appotosis in cultured developing hippocampal neurons. The neurons were exposed one or three times to 6 h of 20, 50, and 100 µM propofol. After treatments, the TUNEL-positive neurons and the protein expression of Cl-Csp3 were analyzed by TUNEL staining (**a**) and western blot (**b**), respectively. Densitometric analysis of Cl-Csp3 protein was performed after normalization with β-actin (**b**). *,^▲^ *P* < 0.05 vs C1 or C2. *C1* control group of single exposure to propofol, *C2* control group of multiple exposure to propofol

The neurons were exposed one and three times to 6 h of 20, 50, and 100 µM propofol. The number of TUNEL-positive neurons in propofol-treated group was significantly increased when compared with these of control group (*P* < 0.05), following one exposure to 50 and 100 µM propofol but not after a single exposure to 20 µM propofol (*P* < 0.05) (Fig. 1a). The number of TUNEL-positive neurons was significantly increased in all propofol-treated groups after three exposures to propofol (*P* < 0.05) (Fig. 1a). Consistently, the protein expression levels of the apoptosis executor cleaved caspase-3 (Cl-Csp3) were also significantly increased by propofol exposure (*P* < 0.05) (Fig. 1b).

### The effects of propofol on tPA release

**Fig. 2:**
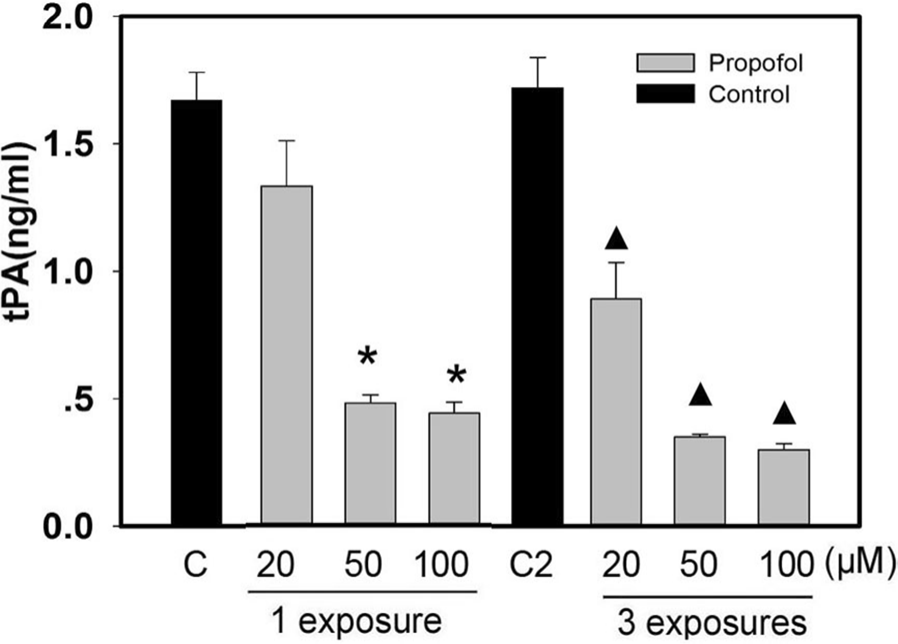
The effects of propofol on tPA release

The neurons were exposed one and three times to 6 h of 20, 50, and 100 µM propofol. The tPA in the media was tested by ELISA. Compared with the control group, neurons exposed to propofol had less tPA in the media (*P* < 0.05) (Fig. 2).

### The effects of tPA addition on propofol-induced appotosis

**Fig. 3:**
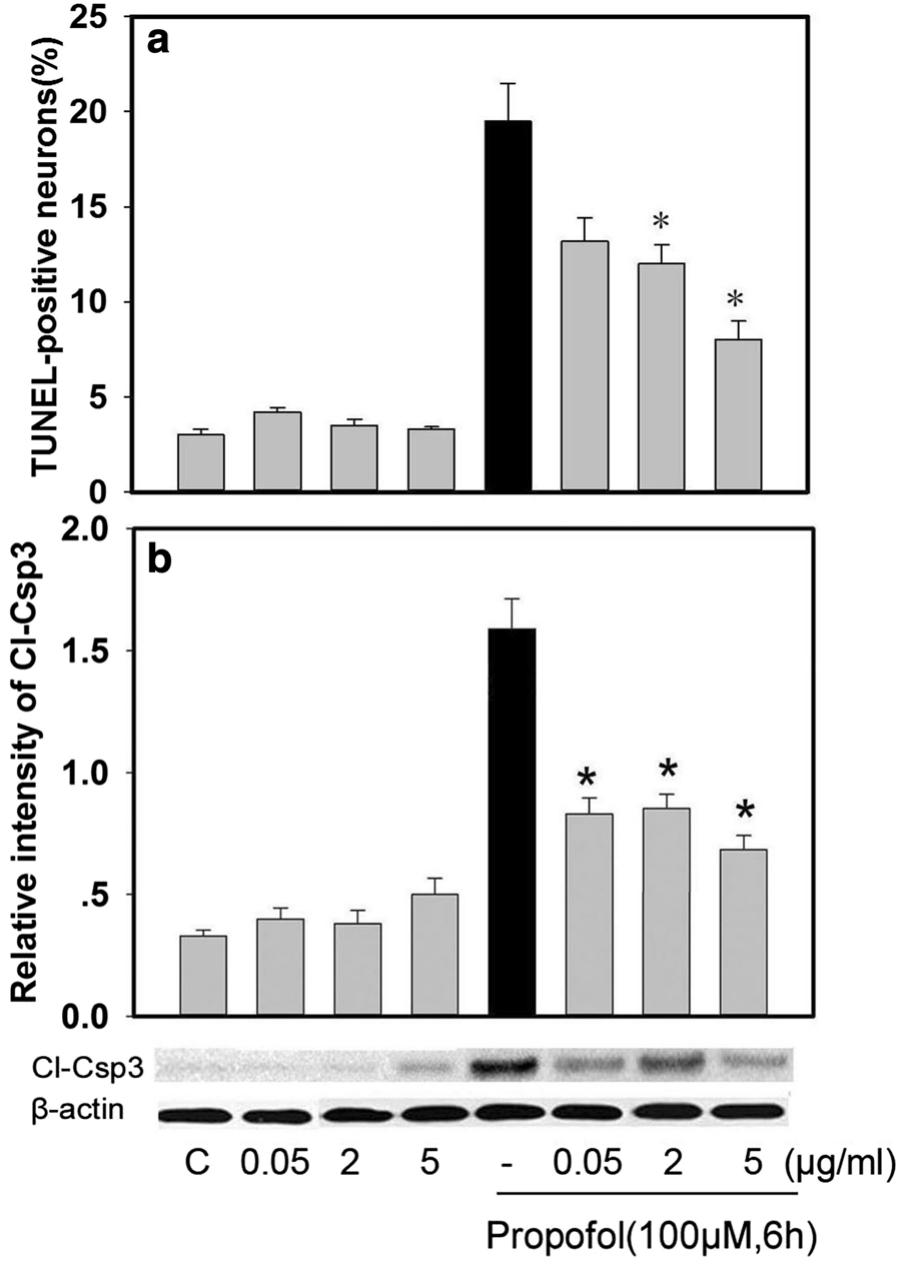
tPA addition attenuates appotosis induced by propofol in cultured developing hippocampal neurons. The neurons were pretreated with increasing doses of tPA (0.05 to 5 µg/ml) before exposed one time to 6 h of propofol 100 µM. After treatments, the TUNEL-positive neurons and the protein expression of Cl-Csp3 were analyzed by TUNEL staining (**a**) and western blots (**b**), respectively. Densitometric analysis of Cl-Csp3 protein was performed after normalization with β-actin (**b**). * *P* < 0.05 vs Propofol group

The neurons were pretreated with increasing doses of tPA (0.05–5 µg/ml) before exposed one time to 6 h of 100 µM propofol. Increasing doses of tPA decreased propofol-induced apoptosis rate and Cl-Csp3 expression with a maximum decrease at 5 µg/ml (*P* < 0.05) (Fig. 3a, b).

## Discussion

The data from the present study indicated that propofol could induce apoptosis in cultured developing hippocampal neurons, which are consistent with the findings from the previous studies (Yu et al. 2013; Pearn et al. 2012; Liu et al. 2014; Twaroski et al. 2014). Importantly, propofol exposure decreases tPA release, and the administration of exogenous tPA attenuates propofol-induced neuron apoptosis.

The new neurons from postnatal hippocampus play an important role in cognitive processes such as learning and memory. It has been reported that anesthetics lead to neurodegeneration during synaptogenesis, which is on the postnatal day 5–7 (Jevtovic-Todorovic et al. 2003).Therefore, in the present study, the isolated hippocampal neurons (postnatal day 1 or 2 pups of rats) were cultured for 4–6 days in vitro, and then undergoing respective treatments. The estimated brain concentrations of propofol in humans during the induction and maintenance of general anesthesia ranges from 22 to 112 μM (Vutskits et al. 2005; Chung et al. 2013; Ludbrook et al. 2002; Costela et al. 1996), and the blood concentration of propofol for the induction of anesthesia and maintenance typically ranges from about 5 to 60 μM in children (Viviand et al. 2003; Varveris and Morton 2002; Hume-Smith et al. 2008).Based on these findings, the propofol concentrations used in our study were 20, 50 and 100 μM, and the neuron apoptosis was prominent at propofol 50 μM, which seems clinically significant. In addition, given propofol was dissolved in DMSO to exclude the potential impact of emulsion, equal volume of DMSO (the final concentration of DMSO was adjusted to 0.005 % for each solution to avoid its possible nonspecific action) was added as the vehicle control in each treatment.

The clinically used tPA (Actilyse^®^) is the approved drug for the acute treatment of ischemic stroke. Unfortunately, the narrow therapeutic window of tPA-induced thrombolysis and the risk of hemorrhage limited its use in clinical practice (Zhang et al. 2011; Saqqur et al. 2008). It also has been reported that tPA might endanger endothelial cells and neurons, resulting in an alteration of the integrity of components of the neurovascular unit (Yepes et al. 2009). In addition, intravenously administerd tPA can induce excitotoxic brain damage in rodents with intact or damaged blood–brain barrier (Benchenane et al. 2005; Lopez-Atalaya et al. 2007). However, the effects of tPA in reversing the neurotoxicity in developing neurons have not been reported in vivo and vitro. On the other hand, the involvement of p75^NTR^ in propofol-induced neurotoxicity has been demonstrated recently, which was verified by p75^NTR^ blockade and knockout (Pearn et al. 2012). Thus,we investgated the effects of propofol on the upstream signals of this pathway. Our data indicated that propofol reduced the amount of tPA present in culture media of developing hippocampal neurons. This reduction in tPA may be due to neuronal suppression by propofol, since tPA release from neurons is in an activity-dependent manner (Baranes et al. 1998; Lochner et al. 2008; Gualandris et al. 1996) and the well-known neuron activity depression effects of general anesthetics. After the inhibiting effects of propofol on tPA release in developing hippocampal neurons was verified, we further found that exogenous addition of tPA can partially reverse the propofol-induced neuron apoptosis.

In addition to direct plasminogen proteolytic function, tPA could also bind to the low density lipoprotein receptor expressed in neurons, which induces the ERK1/2 and Akt mediated antiapoptotic effects (Hu et al. 2008; An et al. 2008). The urokinase-like plasmingoen activator receptor also can be binded by tPA and initiating the intracellular antiapoptotic signaling (Maupas-Schwalm et al. 2004). These findings may indicated that tPA addition not only making the mBDNF-mediated neuron growth promoting pathway dominant, but also initiating the anti-apoptotic effects by other pathways, which need to be elucidated in the future study. Another limitation of our study is that we did not investigate the effects of popofol or the different treatments on the growth of neonatal neurons, such as total number of dendrites, number of primary dendrites, and dendrite length. Moreover, the current study was performed in vitro, whether tPA administration also can attenuate apoptosis induced by propofol in developing neurons in vivo are the purpose of our further study.

## Conclusion

Our study demostrated that propofol decreases tPA release in developing hippocampal neurons, and the addition of tPA could partially reverse the pro-apoptotic effect of propofol. Those findings may project new light on the basis of preventing the propofol-induced neurotoxicity in developing neurons.
